# Two Manners!

**Published:** 1873-02

**Authors:** 


### TWO MANNERS!

Manners for the household, and manners for the public; all sweetness for the stranger, but carping, and complaining, and fault-finding for those at home — the very ones to whom we should look for care and help in case of sickness and dangerous accidents.

Out upon such double characters — such unmanly, such unwomanly, such miserably mean hypocrisies! If you have one spark of generosity, one single ray of nobility of nature left, cherish it as you would an expiring life; kindle it into some holy flame, and come out in the magnanimity of your nature into the sunshine of a more loving heart, of a more kindly countenance, of a more smiling face, and eyes all twinkling with fun and merriment; and our word for it, the sneerish expression will leave the cheek, the hateful snarl will disappear from the voice, the gall-like criticisms will not embitter your utterances, and atrabiliousness and dyspepsias — dyspepsias of the heart as well as those of the stomach — will abate apace, joyous sunshine will dissipate the sombre clouds of the household, and children, and servants, and you yourself will be a thousand times happier. Try it for a week; try for one single week to live without a grunt or a groan, without a snap or a snarl; be more of an angel in spirit, and less like a demon at heart, and may be you will be so pleased with the change that you will ever thereafter try to be

AN ANGEL STILL.

